# Interplay Between Metabolic Pathways and Increased Oxidative Stress in Human Red Blood Cells

**DOI:** 10.3390/cells13232026

**Published:** 2024-12-07

**Authors:** Sara Spinelli, Angela Marino, Rossana Morabito, Alessia Remigante

**Affiliations:** Department of Chemical, Biological, Pharmaceutical and Environmental Sciences, University of Messina, 98166 Messina, Italy; saspinelli@unime.it (S.S.); marinoa@unime.it (A.M.); rmorabito@unime.it (R.M.)

**Keywords:** erythrocytes, redox system, intracellular pathways, metabolism, antioxidant machinery

## Abstract

Red blood cells (RBCs) are highly specialized cells with a limited metabolic repertoire. However, it has been demonstrated that metabolic processes are affected by the production of reactive oxygen species (ROS), and critical enzymes allied to metabolic pathways can be impaired by redox reactions. Thus, oxidative stress-induced alternations in the metabolic pathways can contribute to cell dysfunction of human RBCs. Herein, we aim to provide an overview on the metabolic pathways of human RBCs, focusing on their pathophysiological relevance and their regulation in oxidative stress-related conditions.

## 1. Introduction

Mature red blood cells (RBCs) are relatively simple cells, since they lack most intracellular organelles or internal compartments, yet they serve the important function of transporting and releasing oxygen to the tissues [1]. To accomplish this role, RBCs must travel through capillaries smaller than their own diameter by changing their biconcave shape (deformability) [2,3]. This peculiar plasma membrane property is the result of a composite structure where a plasma membrane envelope is anchored to an elastic network of skeletal proteins through the binding sites of the cytoplasmic domains of trans-membrane proteins placed in the lipid bilayer [4]. RBC cellular functions, energy metabolism, and redox balance are tightly intertwined and dependent on glucose availability [5,6]. In particular, glucose metabolism in human RBCs is essential for (a) ATP production, which is relevant for the plasma membrane and cytoskeleton properties, (b) maintenance of redox balance (NADPH/glutathione), (c) reduction of methaemoglobin to haemoglobin by NADH-reductase, and (d) production of 2,3-bisphosphoglycerate (2,3-BPG), which binds to haemoglobin and releases oxygen [7]. Alterations in the RBC functionality could perturb the metabolism redox balance with an increase in the generation of pro-oxidizing factors up to an oxidative stress condition [8]. However, to cope with higher oxidative stress, RBCs are equipped with a highly efficient redox regulation system strictly committed to maintaining cellular integrity and functionality, represented mainly by enzymes such as superoxide dismutase (SOD), catalase (CAT), glutathione peroxidase (GPx) [9].

Based on current published evidence, RBCs can be an interesting example as a model to examine the relationship between biochemical redox reactions and the maintenance of their physiological properties. Thus, this review aims to summary metabolic pathways of human RBCs, focusing on their pathophysiological relevance and their regulation in oxidative stress-related conditions.

## 2. RBC Metabolic Pathways

### 2.1. Glycolysis

**Figure 1:**
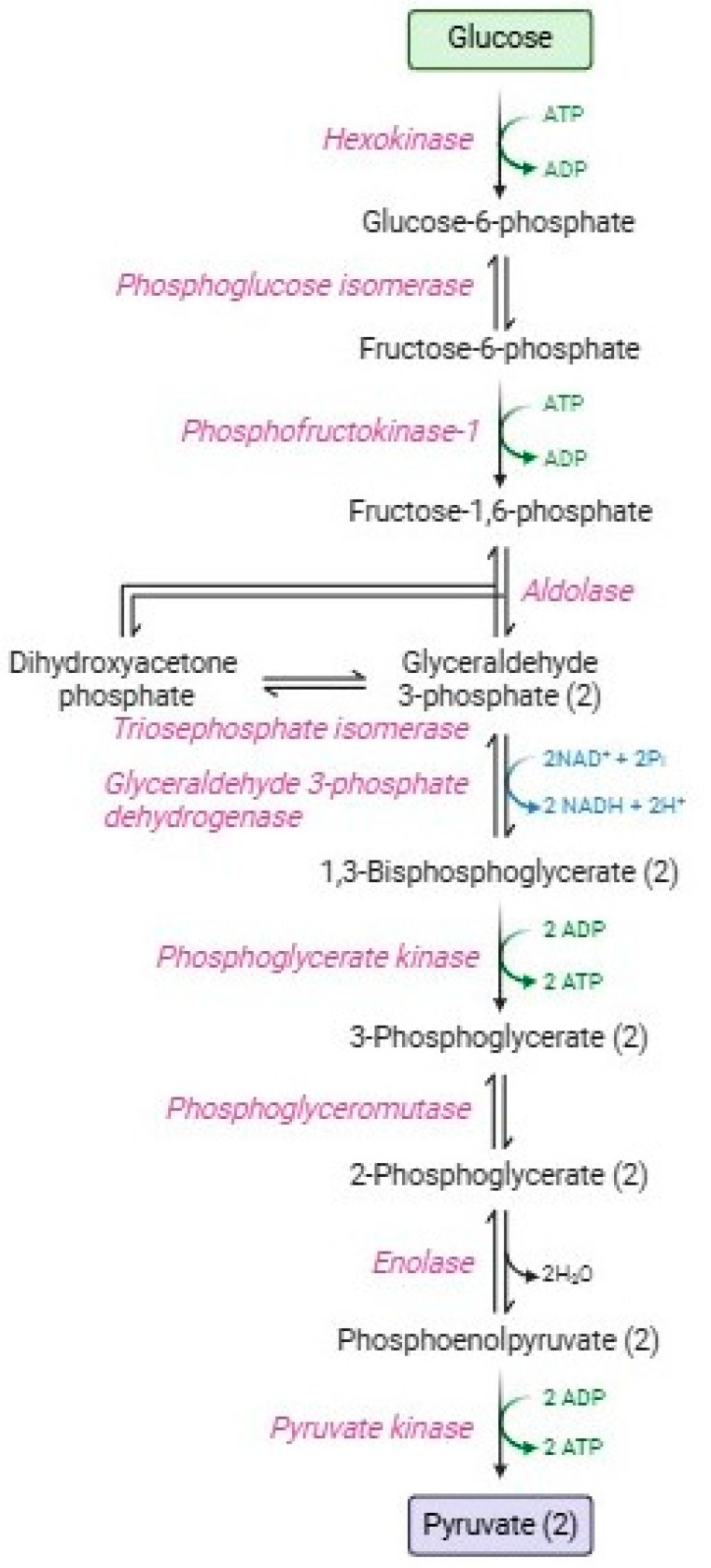
Glycolytic pathway.

Glycolysis or the Embden–Meyerhof–Parnas pathway [10] Figure 1 is a catabolic process consisting of two phases: the energy investment phase and the energy-harvesting phase [11]. In the first phase (or preparatory phase), 90% of glucose is transformed along five enzymatic stages into two glyceraldehyde-3-phosphate molecules, with the consumption of two ATP molecules [12]. The second phase (or payoff phase), is characterized by the other five enzymatic reactions that produce two pyruvate molecules, four ATP molecules, and two NADH, with higher energy production than that employed in the first phase. Red blood cells employ ATP in several homeostatic processes: maintenance of ATPasic pumps (e.g., Na^+^-K^+^/ATPase, Ca^2+^-ATPase, and Mg^2+^-ATPase) [13,14], phosphorylation processes at the level of membrane proteins and lipids, or alternatively, glucose, [15,16,17], haemoglobin allostery [18]; polymerization of cytoskeletal actin [19], vesiculation [20], maintenance of lipid asymmetry by flippases and phosphatidylserine [21,22]; and proteasomic activity to remove damaged proteins [23,24]. Instead, pyruvate is not addressed to the Krebs cycle, but subsequently, it is metabolized by lactate dehydrogenase, resulting in the formation of lactate with the regeneration of NADH [25]. Both pyruvate and lactate can be transported extracellularly through monocarboxylate transporters [26].

Glycolysis has three key regulatory enzymes (aldolase, phosphofructokinase, and glyceraldehyde 3 phosphate dehydrogenase, GAPDH), which constitute an enzymatic multienzyme complex (metabolon) on the inner surface of the RBC membrane [27,28]. These irreversible enzymes have great negative ΔG values essential to guide the overall glycolytic flow [29]. 

**Figure 2:**
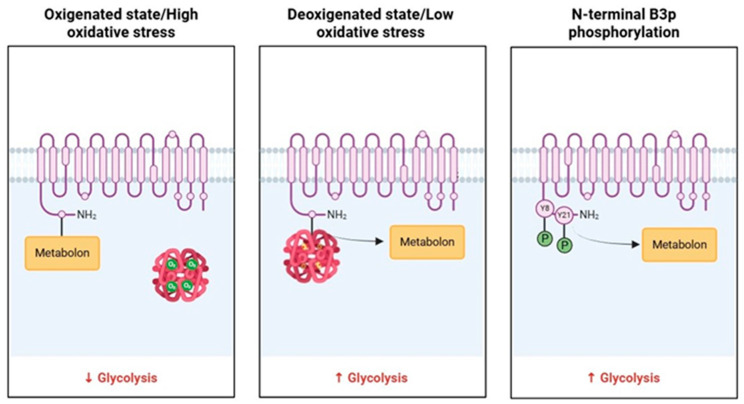
Schematic representation of B3p and metabolon interaction according to the oxygenated state of haemoglobin and B3p N-terminal phosphorylation processes.

The metabolon is a transient structural-functional complex defined as metabolic channeling, whereby the intermediates are maintained within the metabolon, and the reactions are catalyzed by sequential enzymes, thereby regulating metabolic flux through the association and dissociation of the components of the metabolon [30]. Metabolon contributes to the control of glucose metabolism along the glycolytic pathway in association with the RBC band 3 protein (B3p) [27]. The N-terminus cytosolic domain of B3p can bind haemoglobin, cytoskeletal proteins, and glycolytic enzymes [31,32]. The assembly of the metabolon complex on B3p is regulated by the oxygenation levels of haemoglobin and the phosphorylation state of B3p (Figure 2) [27].

The conformational changes induced by haemoglobin deoxygenation (state T) favor its link with B3p [33,34]. Under hypoxic conditions, the haemoglobin-B3p link displaces metabolon enzymes into the cytosol, increasing their activity [31]. Therefore, deoxygenation of haemoglobin promotes glycolysis and 2,3-BPG synthesis by the Rapoport–Luebering shunt to facilitate deoxyhemoglobin stabilization [35,36]. In contrast, haemoglobin oxygenation (R state) is associated with slowing metabolic flow via glycolysis to promote the synthesis of NADPH through the pentose phosphate pathway (PPP) and thus improve antioxidant systems [37,38].

In RBCs, glucose metabolism reacts to complex feedback mechanisms, regulatory enzyme activity, availability of NADH and ATP, and endogenous and/or exogenous stimuli to which they are exposed. Oxidative stress plays a key role in the modulation of glucose metabolism. Specifically, increased ROS causes oxidative damage to glycolysis regulatory enzymes [39]. To name an example, human RBCs exposure to pentachlorophenol, a class 2B human carcinogen, leads to increased ROS levels, thus resulting in the echinocytes and acanthocytes formation, as well as glycolytic and PPP impairment, caused by the lower activity of hexokinase, pyruvate kinase, glucose-6-phosphate dehydrogenase (G6PD) and GAPDH [40]. In addition, this latter enzyme can be highly susceptible to direct ROS action, resulting in the further loss of its own enzymatic activity [41].

Oxidative stress can also promote the formation of disulphide intermolecular bonds between cysteine residues of GAPDH monomers (cysteine 149, 28, and 152), thus leading to enzyme structure alterations, GAPDH aggregation and, ultimately, cellular death [42,43,44]. Therefore, increased oxidative stress can lead to an alteration of glycolysis and a decrease in its products, such as ATP. As a result, low levels of ATP could induce rheological property modification, especially in human RBCs. Metabolic energy depletion can promote echinocytes formation, which leads to a shorter half-life, a greater haemoglobin affinity for oxygen, increased blood viscosity, and ionic concentration imbalance [45]. These conditions occur as a result of pathophysiological processes related to oxidative stress, such as aging and blood storage [46,47]. The close relationship between glycolysis processes and oxidative stress is further demonstrated by the restoration of the glycolytic pathway by antioxidant substances. Lactoferrin, a metal-binding protein with antioxidant activity, counteracts oxidative stress in human RBCs, acting as a metabolic activity regulator. Specifically, such molecule stimulates the activity of glycolytic enzymes, increasing ATP and NAD^+^ synthesis, which is necessary for the maintenance of RBC morphology, membrane potential, and methaemoglobin reductase activity [48]. Alternatively, it has also been demonstrated that ginseng polysaccharide, one of the most bioactive components of *Panax ginseng*, is able to neutralize oxidative damages caused by H_2_O_2_-exposure, thus restoring glycolytic enzyme activity and protecting B3p from degradation [49].

Several studies have shown that increased oxidative stress can lead to oxidation and phosphorylation processes on B3p. These post-translation modifications can induce a re-arrangement of B3p in multiple clusters, possibly following the formation of dimers/oligomers. Moreover, such aggregation could determine the association between molecules of B3p and methaemoglobin, resulting in the production of hemichromes [30,50,51,52]. The oxidation of B3p involves different structural changes that compromise the function of anionic exchanger Cl^−^/HCO_3_^−^ [53,54,55]. In addition, phosphorylation processes of B3p, at the level of tyrosine residues 8 and 21 (operated by Syk kinase) and 359 and 904 (operated by Lyn kinase), provoke an impaired binding between B3p and key glycolysis enzymes, with consequent metabolon detachment and increased glycolytic rate [56], probably as a reasonable defense response to oxidative stress [57]. This process also affects B3p interaction with cytoskeletal proteins, leading to membrane destabilization and the release of microparticles containing hemichromes, as well as demonstrated by several studies on pro-oxidant compounds [58,59,60]. In this context, inhibition of the key glycolysis enzyme function is an effective strategy to determine transient activation of the PPP to generate NADPH and counteract oxidative stress-induced damages [61]. With prolonged oxidative stress, this antioxidant strategy is not effective enough, resulting in irreversible oxidation and phosphorylation not only of functional enzymes but also of structural proteins, such as B3p, ankyrin, and spectrin [62,63], altering the membrane B3p interactome [64].

### 2.2. Pentose Phosphate Pathway (PPP)

**Figure 3:**
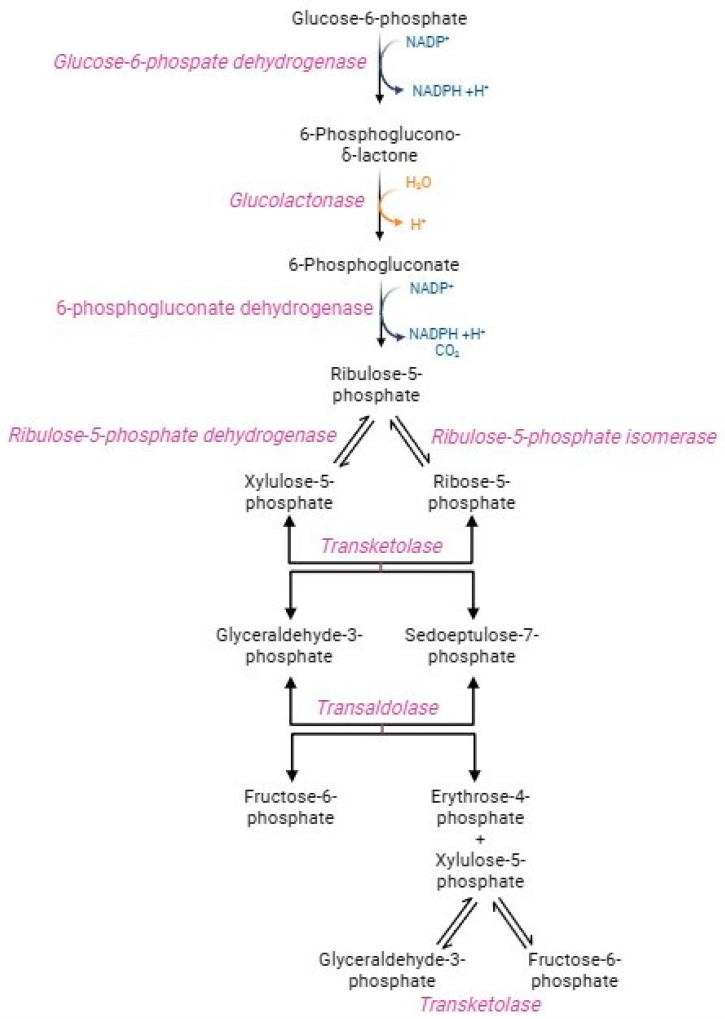
Pentose phosphate pathway.

In physiological conditions, human RBCs metabolize around 90% of glucose through the glycolytic pathway and the remaining 10% via the hexose monophosphate shunt, better referred to as the pentose phosphate pathway (PPP) (Figure 3) [65]. This pathway generates NADPH-reducing equivalents, which transfer electrons in redox reactions rather than energy molecules, such as ATP [65,66]. The oxidative step converts glucose-6-phosphate into ribose-5-phosphate, NADPH, and carbon dioxide. Glucose-6-phosphate dehydrogenase, the PPP first enzyme, catalyzes the oxidation of glucose-6-phosphate to 6 phosphoglucono-γ-lactone, with the reduction of NADP^+^ to NADPH. After hydrolysis of phosphoglucono-γ-lactone to 6-phosphogluconate, another oxidative reaction reduces NADP^+^ to NADPH, with the formation of the pentose ribose-5-phosphate [67]. This phase is very crucial (1) to maintain redox homeostasis in oxidative stress conditions and (2) to trigger the purine salvage pathway for adenine nucleotide production required for ATP synthesis. In parallel, the non-oxidative phase consists of several reactions catalysed by transketolase and trans-aldolase enzymes to obtain sedoheptulose-7-phosphate and two glycolytic intermediates, namely fructose-6-phosphate and glyceraldehyde-3-phosphate, which rejoint the main metabolic stream according to the biochemical demand [67]. Transketolase and trans-aldolase act as a bridge between glycolysis and PPP [66]. The exclusive source of NADPH in human RBCs is PPP, which generates 2 mol NADPH per mol glucose [68]. NADPH drives numerous antioxidant pathways in human RBCs, including the activity of glutathione reductase (GR) and peroxidase 1 (GPX1), peroxiredoxins (PRDX), glutaredoxins, CAT, biliverdin reductase, the ascorbate-tocopherol axis, thioredoxin reductase system, NADPH dependent quinone oxidoreductase, and NAD(P)H-dependent methaemoglobin reductases [7,69]. The activation of PPP is mainly determined by the NADPH/NADP^+^ ratio and the necessity to reduce the NADP^+^ pool [65]. Indeed, the NADP^+^/NADPH ratio regulates the pathway speed-limiting enzyme, namely G6PD [68]. The management of metabolic flows, including glycolysis and PPP, is essential in sustaining the peculiar RBCs metabolic requirements, which depend on oxygen tension and oxidative stress levels [69]. The R state of haemoglobin, in which oxygen tension and affinity increase, leads to improved PPP activity than the T state of haemoglobin [7]. In addition, PPP activity rapidly increases when RBCs are exposed to stressors. Already after the first seconds from the oxidizing event, the glycolysis regulatory enzymes (GAPDH and pyruvate kinase) are inactivated, thus causing glycolysis blockage and glucose diversion to the PPP flow [70,71]. The transition from glycolysis to PPP is a fundamental adaptation mechanism in RBCs, as it facilitates a rapid cellular response that counteracts the increase in stress and oxidative damage [66,72]. In oxidative stress conditions, the demand for NADPH increases and, as a result, PPP activity can rise 20-fold [68,73]. As a result of oxidative insults mediated by the storage of RBCs, the activation of GAPDH was observed [69]. G6PD is essential to counterbalance oxidative stress and, consequently, it could be considered an antioxidant enzyme. The production of NADPH is elicited under oxidative stress conditions as a compensatory mechanism to provide for the increased necessity of reducing equivalents [55,74]. In human RBCs, G6PD deficiency causes sulfhydryl group oxidation, conversion of GSH to GSSG, decreased activity of GPX1 and PRDX2, and displacement of cell redox balance to an oxidized state [75,76]. These processes predispose RBCs to substantial susceptibility to oxidizing agents (such as H_2_O_2_), resulting in NADP^+^/NADPH ratio collapses and, ultimately, haemolysis [37,66,74,77].

### 2.3. Rapoport–Luebering Shunt

**Figure 4:**
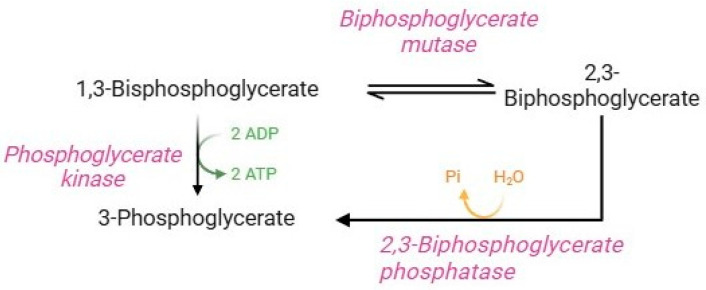
Rapoport–Luebering shunt.

The Rapoport–Luebering shunt (Figure 4) is a parallel glycolysis loop exclusive of human RBCs that represents an important physiological process for regulating several homeostatic functions [12,36]. Glycolytic 1,3-bisphosphoglycerate is shunted to synthesize 2,3-BPG. This reaction is followed by dephosphorylation of 2,3-BPG, which returns to the glycolytic pathway as 3-phosphoglycerate [7]. Quantitatively, 2,3-BPG is the main glycolytic intermediate in RBCs, and its levels are approximately equal to the sum of the other glycolytic intermediates [12]. The diphosphoglycerate mutase, a multifunctional enzyme, is able to catalyze both reactions in the Rapoport–Luebering shunt, and its activity is closely related to pH values. In brief, at basic pH values, synthase activity is promoted (formation of 2,3-BPG via displacement of phosphate from position 1 to position 2 of the molecule). On the contrary, at acidic pH values, phosphatase activity is promoted, which induces hydrolysis of 2,3-BPG to 3-phosphoglycerate [12,78]. 2,3-BPG performs two essential functions in human RBCs: (1) it is necessary to ensure glycolysis efficiency; (2) it is a potent haemoglobin allosteric modulator [36]. Precisely, the binding of 2,3-BPG to haemoglobin decreases haemoglobin’s affinity for O_2_, thus facilitating the release and delivery of O_2_ to peripheral tissues. Specifically, the binding of 2,3-BPG to the β-subunits of deoxyhaemoglobin stabilizes the state T of haemoglobin and thus shifts the oxygen equilibrium curve to the right, promoting oxygen dissociation. In fact, RBCs balance ATP and 2,3-BPG production through diphosphoglycerate mutase activity to ensure RBC homeostasis [7].

**Figure 5:**
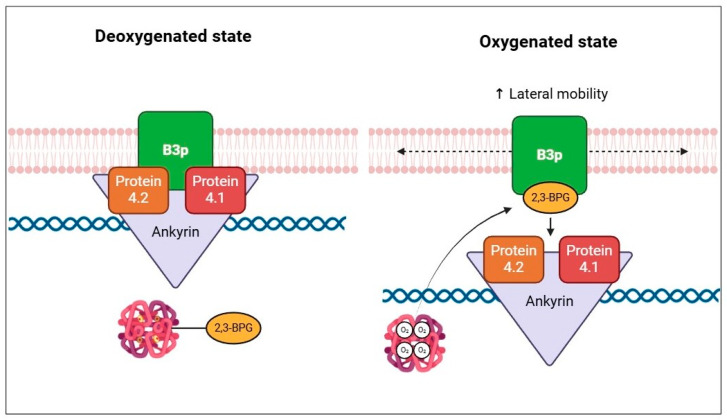
Schematic representation of the effect of 2,3-BPG on cytoskeletal components, according to the oxygenation state of haemoglobin.

In the R state of haemoglobin, about 80% of 2,3-BPGis free, namely unbound to haemoglobin. In this condition, 2,3-BPG modulates the plasma membrane properties of RBCs, binding directly to B3p and thereby impairing the binding to protein 4.1, protein 4.2, and ankyrin [79]. In addition, 2,3-BPG induces spectrin release from the membrane cytoskeleton, negatively interfering in the interactions between spectrin, actin, protein 4.1, and glycophorin C [79]. Decreased interactions between the membrane and cytoskeleton increase the lateral mobility of integral membrane proteins (Figure 5).

As a result, the regulation of RBC levels of 2,3-BPG is critical for effectively supplying tissue oxygen demand, thus providing an important physiological adaptation to hypoxia, a state that underlies several oxidative stress-related diseases [80,81,82]. Under hypoxic conditions, RBCs activate a unique and extremely sophisticated adaptive defense strategy, resulting in the increase of their levels of 2,3-BPG and a reduction of their affinity of haemoglobin for oxygen, thus enhancing its delivery to tissues. Such a strategy, demonstrated by Chen and co-authors [82], interconnects the Rapoport–Luebering shunt with purine metabolism. This compensatory response involves different players, including adenosine, ENT-1 (equilibrative nucleoside transporter 1), AMPK (AMP-activated protein kinase), AMPD3 (AMP-deaminase 3), and diphosphoglycerate mutase. In hypoxic conditions, extracellular adenosine accumulates within RBCs via ENT-1, and this latter is phosphorylated to AMP by AMPK. Increased AMP, coupled with AMPD3 inhibition, induces activation of diphosphoglycerate mutase, leading to the production of 2,3-BPG. Thus, RBCs perform an AMPK-BMPG-dependent metabolic reprogramming by exploiting the two main sensors of hypoxia (ENT-1 and AMPD3) to cope with the increased oxygen demand and mitigate the oxidative stress induced by hypoxia-related diseases.

### 2.4. Carboxylic Acid Pathway

**Figure 6:**
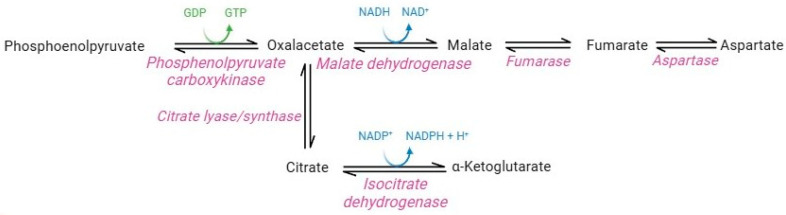
Carboxylic acid pathway.

Although lacking nuclei and organelles, mature RBCs are considerably more complex than was believed until the last decade. Originally, the absence of mitochondria and their enzymes precluded the possible presence of carboxylic acid metabolism in this cellular system [7]. However, proteomics studies instead revealed the presence of cytosolic isoforms of Krebs cycle enzymes in mature RBCs (Figure 6) [83]. In particular, functional cytosolic isoforms of phosphoenolpyruvate carboxykinase, malate dehydrogenase, fumarate hydratase, aspartase, isocitrate lyase/synthase, and isocitrate dehydrogenase have been identified [37].

The question concerned their potential activity and their presumably influence on the entire RBC metabolism. Pyruvate and citrate catabolism reactions operated by the cytosolic isoforms of Krebs cycle enzymes may contribute to the production of the NADH and NADPH-reducing equivalents in RBCs [83]. Such production is promoted in several pathophysiological conditions that share increased oxidative stress. For example, a study performed by Nemkov and co-authors [83] showed that RBC exposure to hypoxia promotes the catabolism of carboxylic intermediates, such as citrate and malate, through the activity of isocitrate dehydrogenase and malate dehydrogenase, thus stimulating the production of NADPH and NADH. Moreover, significant increases in carboxylic acid cycle intermediates (succinate, fumarate, and malate) were observed in G6PD-deficient RBCs. Due to the decreased capability to produce NADPH through PPP, RBCs probably use this metabolic pathway as a compensatory mechanism [84]. Overall, these studies demonstrate the extraordinary adaptive capacity of RBCs, which could switch their metabolism according to physiological needs and external conditions.

### 2.5. Purine Salvage Pathway

**Figure 7:**
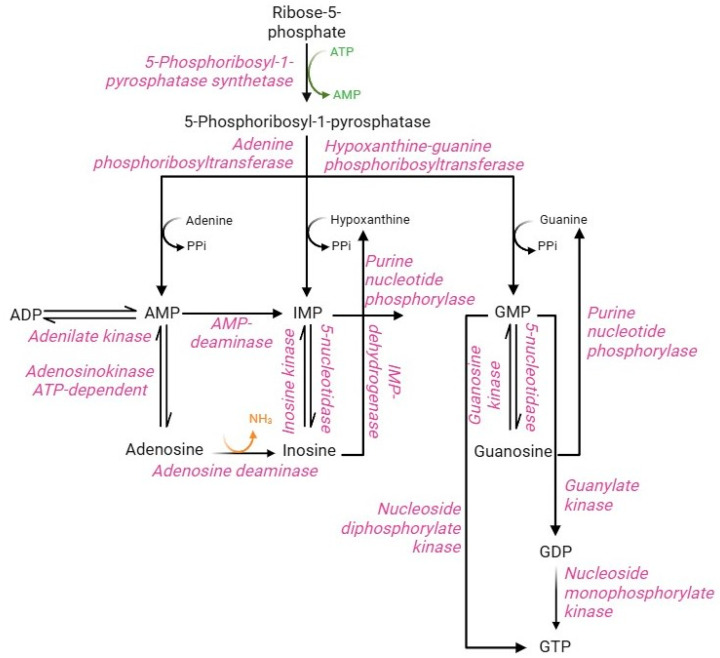
Purine salvage pathway.

Purine metabolism involves synthesis and degradation processes of purine nucleotides to generate an intracellular pool of ATP and GTP. RBCs cannot synthesize purine nucleotides de novo, due to the lack of the key enzyme, namely glutamine 5-phosphoribosyl-1-pyrophosphate (PRPP) aminotransferase [85]. Therefore, in RBCs, the purine bases (adenine, hypoxanthine, and guanine) from the bloodstream and the nucleosides (adenosine, inosine, and guanosine) are metabolized and reutilized via the purine salvage pathway [86]. The purine salvage pathway (Figure 7) has profound physiological relevance in RBCs, allowing the production of the energetic equivalents despite the absence of the enzymatic component. This can be accomplished by interconnecting this pathway with PPP and glycolysis. In summary, PPP supplies, via ribose-5-phosphate through the action of PRPP synthase enzyme, the main metabolic substrate of the purine salvage pathway, namely phosphoribosyl-1-pyrophosphatase, while glycolysis supplies specific enzymes, such as phosphofructokinase and phosphoglycerate kinase, able to convert the ADP into ATP [85]. Their synthesis is regulated by multiple factors, including the availability of ribose-5-phosphate, the content of reaction products, and the concentration of 2,3-BPG, which accumulation leads to monomerization of the PRPP synthase enzyme resulting in loss of enzyme activity [5]. Such metabolite represents an essential precursor for the conversion of adenine, hypoxanthine, and guanine into monophosphorylated purine nucleotides: AMP, IMP, and GMP [85,87]. The production of AMP, IMP, and GMP can likewise occur from nucleosides (adenosine, inosine, and guanosine, respectively) by kinase enzymes with different specificity for the three substrates. In turn, monophosphorylated purine nucleotides produce their respective nucleosides by dephosphorylation reactions mediated by adenosine kinase (AMP to adenosine) and 5-nucleotidase (IMP to inosine and GMP to guanosine) enzymes [88]. AMP, produced by the purine salvage pathway, can be converted to ADP by the adenylate kinase enzyme. Instead, ADP can be converted to ATP by the glycolytic enzymes (phosphoglycerate kinase and pyruvate kinase); both are the only RBC reactions that allow ATP synthesis [5]. Similarly, GMP can generate GDP by nucleoside monophosphate kinase-mediated phosphorylation or GTP by nucleoside dephosphorylate kinase. Alternatively, GDP can be phosphorylated to GTP by guanylate kinase [89]. In contrast to other cell types, IMP cannot be converted to AMP because of the lack of the enzyme adenyl-succinate synthase [90]. Therefore, the deamination reaction of AMP to IMP by the enzyme AMP deaminase will be favored. The IMP pool is essential to produce GMP, which occurs in two steps by IMP dehydrogenase enzyme in addition to the dephosphorylation of guanine. In physiological conditions, the total content of intra-erythrocytic AMP, IMP, and GMP is constantly maintained despite the possible alteration of nucleotide pool levels. This metabolic stability is ensured by the processes of resynthesis, interconversion, and degradation of purine compounds, as well as by the regulatory mechanisms of the different enzyme activities [85].

Oxidative stress profoundly affects the balance between purine bases, nucleosides, and phosphorylated nucleotides of the purine salvage pathway. Indeed, ATP synthesis is decreased for two main reasons: the oxidative sensitivity of glycolytic enzymes that mediate ATP synthesis (phosphofructokinase and pyruvate kinase) and the binding of these enzymes to B3p [91,92]. In this context, ATP is cleaved into ADP and AMP, and the latter becomes a substrate for deamination to IMP by AMP deaminase. IMP undergoes phosphoribolysis, releasing hypoxanthine, which is oxidized by xanthine oxidase. This enzyme produces xanthine, urate, and H_2_O_2_ [7,93]. The increase in oxidative stress is exacerbated by the deamination of adenosine to inosine by adenosine deaminase, which contributes to the synthesis of hypoxanthine by the action of purine nucleoside phosphorylase. In this context, abnormal levels of deaminated purines were associated with haemolysis and morphological changes in the storage of RBCs [5]. In parallel, Tavazzi and co-workers [94] has reported (a) ATP depletion, (b) AMP deaminase activation, (c) increased IMP levels, and (d) efflux of inosine, hypoxanthine and xanthine in human RBCs treated with increasing concentrations (0.5–10 mM) of H_2_O_2_. Moreover, metabolic changes, increased levels of lipid peroxidation, haemolysis were also reported. In contrast, as discussed above, hypoxia exploits the purine salvage pathway to trigger the adaptive response of RBCs to oxygen deprivation. Specifically, hypoxic conditions prevent purine oxidation via inhibition of AMP deaminase and promote purine salvage reactions via activation of AMP kinase. This response is mediated by extracellular adenosine uptake via ENT-1. In RBCs exposed to pyridamole (a selective inhibitor of ENT-1), low adenosine levels lead to aberrant ROS-dependent AMP deaminase activation, resulting in impaired energy metabolism, hypoxia-induced lesion, and chronic kidney disease [82,95].

### 2.6. Hexosamine Pathway

**Figure 8:**
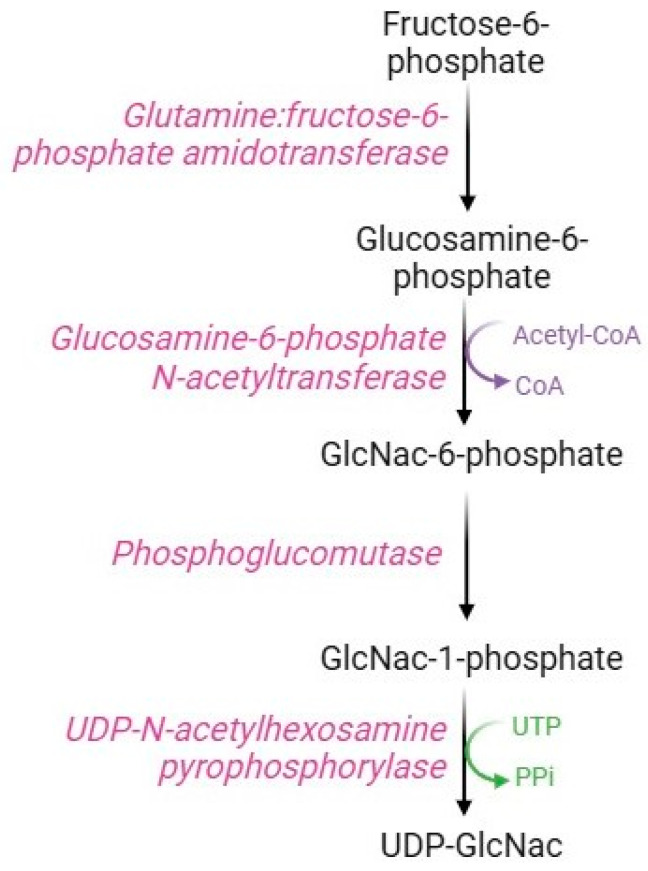
Hexosamine pathway.

Although most of the intracellular glucose is metabolized by the glycolytic pathway, some of the fructose-6-phosphate is used in the hexosamine pathway (Figure 8), a metabolic route devoted to the synthesis of substrates necessary for glycosylation processes. The key enzyme in this pathway is glutamine: fructose-6-phosphate amido-transferase, which uses glutamine and fructose-6-phosphate as substrates to produce glucosamine-6-phosphate. Specifically, this rate-limiting enzyme possesses an N-terminal glutaminase domain that catalyzes the hydrolysis of glutamine to glutamate and ammonia and a C-terminal isomerase domain that uses ammonia to convert fructose-6-phosphate to glucosamine-6-phosphate [96]. Subsequently, the enzyme glucosamine-phosphate N-acetyltransferase transfers an acyl group to glucosamine-6-phosphate to form N-acetylglucosamine-6-phosphate (GlcNAc-6-P), which is converted to N-acetylglucosamine-1-phosphate (GlucNAc-1-P) via the action of phosphoglucomutase [97]. The last step of this pathway consists of the conversion of UTP and GlcNAc-1P to UDP-GlcNAc and pyrophosphate catalyzed by UDP-N-acetyl-hexosamine pyro-phosphorylase [98].

UDP-GlcNac is a vital metabolite employed as a substrate for the production of glycosyl side chains in the post-translational modifications of target proteins [99]. This dynamic process is named GlucNAcylation [100], which consists of the GlucNAc moiety binding to the hydroxyl group of serine and/or threonine residues (O-GlucNAcylation), or alternatively, to the amino group of asparagine (N-GlucNAcylation) of cytoplasmic or nuclear proteins [101,102]. O-GlcNAcylation is regulated by two key enzymes, O-GlucNAc transferase (OGT) and O-GlcNAcase (OGA), which mediate the α-O-glycosidic bond formation or disruption, respectively [103]. On the contrary, N-GlcNAcylation is regulated by the enzyme oligosaccharyltransferase, which targets the consensus Asn-X-Ser/Thr sequence on the polypeptide chain and establishes the β-1,4-glycosidic bond [104]. The O-GlcNAcylation process plays important physiological functions, such as regulation of intracellular signaling, gene transcription, and maintenance of structural integrity of cells and tissues [96,102]. At the same time, the process of N-GlcNAcylation is very important to ensure proteostasis by mediating the proper protein folding, stability, and function [102]. The hexosamine pathway physiologically acts as a nutrient sensor: under normoglycemic conditions, a small amount of fructose-6-phosphate is shunted into this pathway, ensuring adequate glycosylation processes [102]. However, in RBCs, O-GlucNAcylation is also able to modulate the necroptotic process via post-translational modifications of pre-existing proteins [105]. Indeed, O-GlcNAcylation of RIPK1 (receptor-interacting protein kinase1) serine 331 inhibits the phosphorylation of RIPK1 on serine 166, which is required for the formation of the RIPK1-RIPK3 complex with necroptotic (membrane destruction) activity.

As previously demonstrated [106,107], GlcNAc levels significantly increase as a result of several forms of stress (heat, UV, hypoxia, oxidative, and osmotic stress) and recover to basal levels after 24–48 h. This temporary increase could be attributed to an increased glucose flux into the cells, potentially triggered by a stress condition, or alternatively, as a result of increased OGT activity or decreased OGA activity [108]. The balance of this pathway may be disrupted in different diseases related to an increase in oxidative stress levels. To name an example, increased hexosamine pathway activity may mediate the toxic effects of ROS in hyperglycaemia and insulin resistance; both are the main hallmarks of type 2 diabetes [109]. In this regard, increased blood glucose concentration induces over-shunting of fructose-6-phosphate in the hexosamine pathway, resulting in hyperactivity of the enzyme glutamine:fructose-6-phosphate amido-transferase [110]. The subsequent accumulation of UDP-GlucNAc triggers GlcNAcylation processes associated with the pro-oxidant effects of the hexosamine pathway [111]. However, specific inhibitors of the enzyme glutamine:fructose-1,6-biphosphate amido-transferase are able to abrogate the cellular effects of hyperglycaemia [112,113]. Paradoxically, it has been demonstrated that activation of the hexosamine pathway can elicit adaptive cellular responses in oxidative stress-related diseases. For example, in RBCs of pre-diabetic or diabetic subjects, O-GlcNAcylation processes are increased in order to ensure the stability and functionality of target proteins [114,115]. As demonstrated by Ruan and colleagues, O-GlcNAcylation modifications can protect proteins from degradation caused by competitive action with the phosphorylation processes required to regulate ubiquitination [116]. When the stressful condition is alleviated, O-GlcNacylation levels can return to basal values by the OGA enzyme. Then, modulation of O-GlcNAc levels is a key mechanism in the adaptive response to cellular stress. In brief, reduced levels of OGT and O-GlcNAc result in impaired stress tolerance; on the contrary, increased levels of OGT and O-GlcNAc render cells more tolerant. Considering the complexity of such a metabolic pathway, future studies are needed to clearly understand the mechanisms underlying the paradoxical effect of the hexosamine pathway.

### 2.7. Arginine Pathway

**Figure 9:**
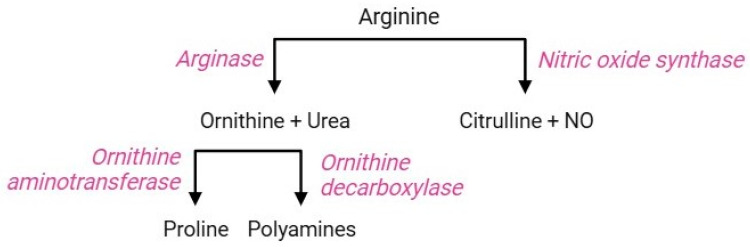
Arginine pathway.

The arginine pathway (Figure 9) includes a sequence of parallel reactions that occur in all cells. The occurrence of the key functional enzymes of this pathway has been established both in erythropoietic precursors and mature RBCs [117]. In the former, the arginine pathway contributes to the regulation of intracellular pH during the hematopoietic process [118]; in the latter, this pathway triggers cellular responses as a consequence of iron-deficiency anaemia or abiotic stresses. The RBC membrane is equipped with cationic amino acid transporters for L-arginine, L-lysine, and L-ornithine, referred to as CAT1, CAT2a, and CAT2b; among them, CAT1 is the main transporter of L-arginine in RBCs [119]. Once delivered to the intraerythrocytic environment, arginine becomes a substrate for two enzymes: arginase 1, which performs a proteolytic cleavage of the guanidine group to form ornithine and urea, or alternatively, nitric oxide (NO) synthase converts arginine to citrulline and NO [120]. The regulatory mechanism leading to the activation of either competitive enzyme has not yet been elucidated, but the negative correlation between arginase activity and NO synthesis is known [119]. Ornithine, produced by arginase 1, can be decarboxylated by sequential reactions through the action of ornithine decarboxylase, producing different polyamines (spermine, spermidine, and putrescine) [121]. Alternatively, ornithine can also be metabolized by ornithine aminotransferase to form proline, which is essential for collagen production and maintenance of intracellular redox homeostasis [122,123].

In RBCs, the occurrence of two functional isoforms of NO synthase (endothelial and inducible; eNOS and iNOS, respectively) has been identified [124], which produce NO. Physiologically, NO performs several autocrine and paracrine functions, such as increased RBC deformability (which promotes RBC transit in narrow capillaries), decreased aggregation of platelets, cell adhesion inhibition, and vascular smooth muscle vasodilation [125,126]. Both arginase and NO synthase reactions crosstalk with other RBC metabolic pathways, such as glutathione homeostasis and heme synthesis of haemoglobin [7].

Despite a plethora of primary physiological functions, several studies have shown an imbalance between arginase and NOS activity in oxidative stress conditions. Specifically, increased ROS upregulates arginase activity [127], which consumes arginine. In this condition, NOS is deprived of its substrate but retains its ability to donate electrons from NADH [119,128]. The main acceptor of these electrons is oxygen, which is oxidized to superoxide anion (O_2_^.−^) and reacts with NO to form peroxynitrite (ONOO^−^) [129]. Peroxynitrite performs a double action: (1) it fuels the already existing oxidative stress; (2) it oxidizes tetrahydrobiopterin, a NOS cofactor, leading to further uncoupling of NOS and aggravation of the oxidative condition [120,130]. In addition, decreased NO triggers inflammatory processes via increased platelet aggregation and leukocyte attachment to vascular endothelium [123]. In such an unfavorable scenario, increased arginase activity leads to the overproduction of polyamines, which form ROS as by-products. For example, spermine is oxidized by spermine oxidase, producing H_2_O_2_ and acrolein, an aldehyde that induces increased RBC membrane stiffness, increases osmotic sensitivity, and promotes eryptosis [123]. Oxidative stress-induced arginine metabolism alteration has been found in several diseases. In particular, a correlation between arginase activity and endothelial dysfunction has been demonstrated in RBCs obtained from diabetic patients [129,131]. However, in RBCs treated with 2-(S)-amino-6-boronohexanoic acid (a specific arginase inhibitor), or alternatively, with 20-tetrakis(4-sulfonatophenyl) porphyrinate chloride (a peroxynitrite scavenger) resulted in a complete recovery of endothelial dysfunction [132]. Arginase activity is also found to be increased in RBCs obtained from patients with chronic obstructive pulmonary disease, an oxidative stress-related inflammatory disorder induced by chronic exposure to tobacco smoke [133]. The interdependence between arginase activity and oxidative stress yields this enzyme an excellent indicator of the health status of subjects exposed to toxic substances. Indeed, a comparative study performed by Fukumoto and colleagues [134] demonstrated increased arginase activity in RBCs in response to occupational lead exposure and a dose-response correlation.

### 2.8. Lands Cycle

**Figure 10:**
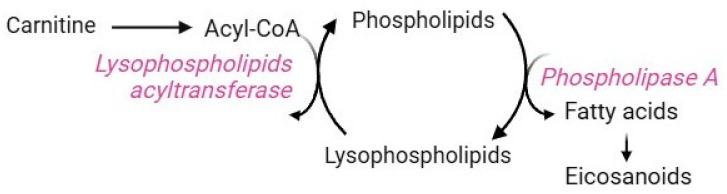
Lands cycle.

The Lands cycle is a key biochemical process that remodels the plasma membrane to control both the composition and positional specificity of fatty acyls in cellular and tissue phospholipid pools [135]. Mature RBCs use this pathway to counteract the damages caused by increased lipid peroxidation. Their plasma membrane is very rich in polyunsaturated fatty acids, whose double bond structure makes them more prone to oxidation than other lipids [136,137,138]. Membrane peroxidation may lead to a reduction in RBC deformability and eryptosis [5]. Due to the lack of Golgi and endoplasmic reticulum, RBCs do not synthesize phospholipids de novo [139] but take up fatty acids from the bloodstream [140]. Therefore, maintaining and renewing membrane lipids are essential processes to ensure RBC homeostasis. These tasks are exclusively accomplished by the Lands cycle, which is mediated by two enzymes that work in concert: phospholipase A2 (PLA2) and lysophospholipid acyltransferases (LPLATs) (Figure 10) [139]. In brief, the phospholipid sn-2 ester bond is specifically hydrolyzed by PLA2, which releases the oxidized fatty acid moiety, thus generating lysophospholipids. Subsequently, LPLATs transfer the acyl-CoA acyl group derived from carnitine to lyso-phospholipids, thus, regenerating phospholipids. At this point, the de-acylation/re-acylation repair reactions are complete [139]. Clearly, the availability of carnitine limits the rate of this pathway [7]. Dysregulation of the Lands cycle is observed under oxidative stress conditions, which induce alterations in the fatty acyl composition of the RBC membrane due to a disruption in lysophospholipid metabolism [140]. For example, an abnormality in membrane lipid composition and organization has been reported in sickle RBCs, making them more susceptible to lipid peroxidation [139]. In this context, the Lands cycle contributes to sickling, inflammation, and tissue damage by promoting the accumulation of lysophospholipids due to the overactivity of PLA2 and the failure of LPLATs to recycle them. Lysophospholipids are bioactive molecules that may interact with plasma membrane components, thus altering membrane shape, permeability, and trafficking events [141].

### 2.9. Glutathione Pathway

**Figure 11:**
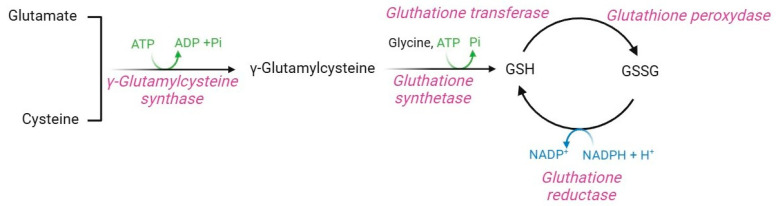
Glutathione pathway.

Glutathione (GSH, γ-glutamyl-L-cysteine) is a non-enzymatic regulator of intracellular redox homeostasis that represents one of the major antioxidants synthesized by RBCs [5]. GSH half-life is approximately 3–4 days, thus, it is constantly produced within RBCs [65,142]. GSH is synthesized from the precursor amino acids cysteine, glutamic acid, and glycine via two ATP-dependent enzymatic reactions (Figure 11). The first is catalyzed by γ-glutamylcysteine synthase, which forms an isopeptide bond between glutamic acid and cysteine to produce γ-glutamylcysteine. A second reaction, catalyzed by GSH synthase, leads to the binding of glycine to γ-glutamylcysteine, thus forming GSH [143]. GSH synthase is a heterodimeric holoenzyme complex that comprises a catalytic subunit, which contributes to its enzymatic action, and a regulatory subunit, which modulates the catalytic subunit activity and the substrate affinity [144,145].

Regulation of this pathway depends on amino acid availability, particularly cysteine, ATP levels, and negative feedback mechanisms mediated by GSH levels [143,146,147]. In human RBCs, GSH is both synthesized and recycled through NADPH-dependent GSH reductase, which mediates the conversion of oxidized glutathione (GSSG) to GSH [148]. The GSH protective mechanism is achieved through the amino acid cysteine, which possesses a thiol group (-SH) useful for detoxifying electrophilic reactive molecules [149]. Hence, GSH acts as a direct ROS scavenger, a substrate of the glutathione peroxidase [5], a vitamin C recycling agent [150], and a protein protector via the glutathionylation (GS-silylation) reaction [151]. GS-silylation is a post-translational modification that involves the attachment of a GSH molecule to a cysteine residue of a target protein. This process, which may occur spontaneously or through the glutathione-S-transferase enzyme, modulates protein function, inhibits or enhances enzymatic activity, maintains redox homeostasis, and protects several proteins from irreversible oxidative damage [152]. In human RBCs, GS-silylation occurs at the haemoglobin level: in physiological conditions, GSH binds to the C93 residue of haemoglobin, close to the histidine residue involved in iron coordination within the heme group. This binding increases the solubility of haemoglobin, which thus acts as an intracellular GSH reservoir and a regulator of RBC redox homeostasis [37,153]. Proper intracellular GSH levels are critical for the regulation of cell metabolism via oxidation-reduction reactions triggered by the -SH group of GSH cysteine, which serves as a reducing agent [154]. GSH is thus the main coenzyme of several enzymatic defense systems, such as glutathione-S-transferase. This latter is essential for detoxifying toxic xenobiotics, to which GSH covalently binds to form water-soluble conjugates exported from RBCs for excretion, thus shedding the oxidative burden [145]. In this context, Perrone and colleagues [155] have reported that the exposure of RBCs to mercury, a toxic heavy metal widely distributed in the natural environment, provokes a GSH/GSSG ratio decrease due to the binding between this xenobiotic and the -SH group of GSH. To fulfill all these roles and provide protection from oxidation, intracellular GSH levels should be around 2.2 µmol/mL RBCs, and the GSH/GSSG ratio should be greater than 500. These parameters ensure the protection of haemoglobin -SH groups from disulphide cross-linking with other cytoplasmic proteins [65].

As mentioned above, the maintenance of adequate levels and turnover rates of GSH is required for several cellular functions, and defects in these processes are observed in many human pathologies [156]. GSH deficiency is primarily manifested by an increased susceptibility to oxidative stress. At the same time, excessive GSH levels cause a condition known as “reductive stress”, namely an imbalance between the oxidant species levels and the reducing capacity of cells in favor of the latter [157]. Reductive stress induces a decline in ROS content below physiological levels, thus disrupting their signaling function, cell metabolism, and protein disulphide bridge formation. Paradoxically, reductive stress also promotes increased oxidative stress, e.g., by partially reducing oxygen with a ROS generation [158,159]. Oxidative stress per se or derived from reductive stress affects GSH redox homeostasis, rendering some RBC components, such as haemoglobin, the major target of oxidative damage. To confirm this, a genetic deficiency of the enzymes γ-glutamyl-cysteine synthase and glutathione synthase results in the oxidation of cysteine residues G14 and G11 of haemoglobin, leading to the dissociation of its tetrameric structure into monomers [65]. The precipitation of these monomers causes the formation of insoluble hemicromes, which may bind B3p via the formation of disulphide bridges, generating Heinz bodies with consequent haemolytic anaemia [50,65,160].

### 2.10. Glutathione Peroxidase 4 (GPX4) Pathway

**Figure 12:**
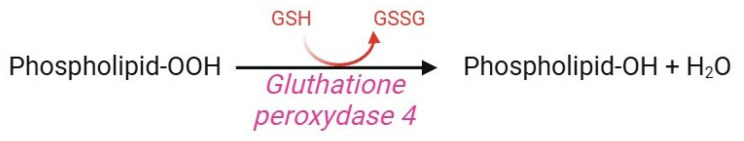
Glutathione peroxidase pathway.

Glutathione peroxidase (GPx) 4 is a selenoenzyme member of the GPx family, which also includes GPx1, GPx2, and GPx3. Notwithstanding, GPx4 does not degrade H_2_O_2_, alkyl peroxide, or fatty acid hydroperoxide, but rather hydroperoxide of lipoproteins and complex lipids in biological membranes, such as cholesterol, cholesterol esters, and phospholipids (Figure 12) [161]. To accomplish this task, GPx4 contains the amino acid selenocysteine instead of cysteine in its active site, which makes it more resistant to irreversible hydroperoxide-mediated oxidation [162]. GPx4 is a key component of the cellular antioxidant system since it is the only enzyme able to prevent membrane lipid peroxidation via the following mechanism. In human RBCs, the presence of GPx4 (confirmed by proteomic analysis [163]) is essential to preserve redox homeostasis through the GS-nylation process [7,164]. A reduction in GPx4 concentration or activity, as well as a genetic polymorphism in the coding region of the *GPx4* gene, is associated with an increased rate of lipid peroxidation in oxidative stress conditions or alternatively, during RBC storage [165]. Interestingly, GPx4 abundance is 75% heritable and exhibits at least four-fold variation between cohorts of donors, predisposing their RBC to increased susceptibility to haemolysis [166].

## 3. Conclusions

**Figure 13:**
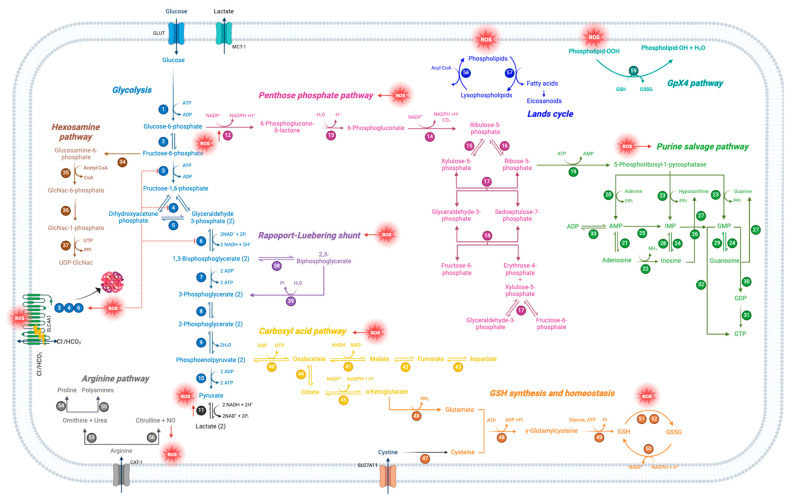

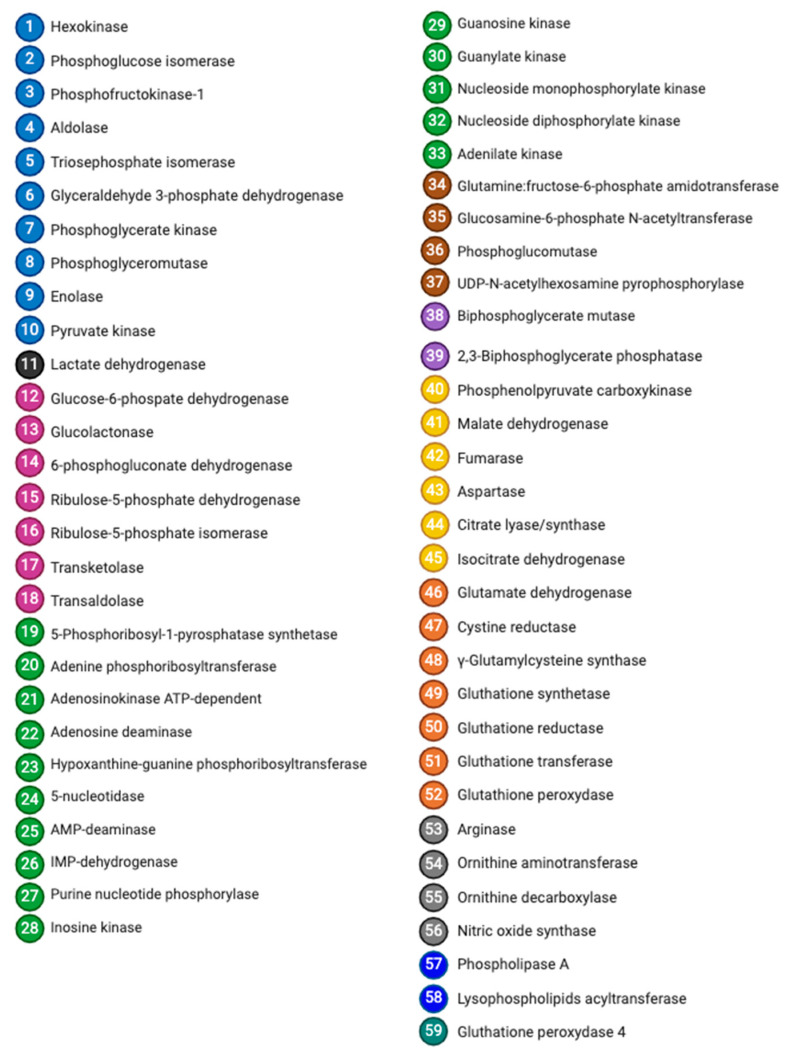
Schematic representation of oxidative stress-modulated metabolic pathways in human RBCs. Each number represents a specific enzyme involved in the pathway.

Mature RBCs, losing almost all cytoplasmatic organelles, only conserve a few metabolic pathways for obtaining energy and, in parallel, reduce the energy consumption for the key functions they need to fulfill. This makes RBCs highly sensitive to any disorder. As reported above, several investigations have explored in detail the impact of oxidative stress on RBC metabolism (Figure 13). Anyway, despite the advances of the last half-century, further study is clearly warranted to decipher the precise oxidative stress-related molecular mechanisms underlying an altered energetic metabolism. However, since these cells can sense blood changes early and continuously, metabolic indicators related to RBCs can provide more clinical information and can be used to monitor the progression of specific diseases and their complications.

## Data Availability

No new data were created or analyzed in this study. Data sharing is not applicable to this article.
